# The Role of Bioactive Dietary Components in Modulating miRNA Expression in Colorectal Cancer

**DOI:** 10.3390/nu8100590

**Published:** 2016-09-26

**Authors:** Laura I. Gavrilas, Corina Ionescu, Oana Tudoran, Cosmin Lisencu, Ovidiu Balacescu, Doina Miere

**Affiliations:** 1 Department of Bromatology, Hygiene, Nutrition, University of Medicine and Pharmacy “Iuliu Hatieganu”, Marinescu Street 23, Cluj-Napoca 400337, Romania; laura.biris@umfcluj.ro (L.I.G.); dmiere@umfcluj.ro (D.M.); 2 Department of Pharmaceutical Biochemistry and Clinical Laboratory, University of Medicine and Pharmacy “Iuliu Hatieganu”, Louis Pasteur Street 6, Cluj-Napoca 400349, Romania; inaionescu@yahoo.com; 3 Department of Functional Genomics, Proteomics and Experimental Pathology, The Oncology Institute “Prof. Dr. Ion Chiricuta”, Republicii Street 34-36, Cluj-Napoca 400015, Romania; oana.tudoran@iocn.ro; 4 Department of Surgical and Gynecological Oncology, University of Medicine and Pharmacy “Iuliu Hatieganu”, Republicii Street 34-36, Cluj-Napoca 400015, Romania; cosminlisencu@yahoo.com; 5 Department of Surgery, The Oncology Institute “Prof. Dr. Ion Chiricuta”, Republicii Street 34-36, Cluj-Napoca 400015, Romania

**Keywords:** colorectal cancer, microRNA, diet, bioactive dietary components

## Abstract

Colorectal cancer is the third most common cancer in the world and considered to be one of the most diet-related types of cancer. Extensive research has been conducted but still the link between diet and colorectal cancer is complex. Recent studies have highlight microRNAs (miRNAs) as key players in cancer-related pathways in the context of dietary modulation. MicroRNAs are involved in most biological processes related to tumor development and progression; therefore, it is of great interest to understand the underlying mechanisms by which dietary patterns and components influence the expression of these powerful molecules in colorectal cancer. In this review, we discuss relevant dietary patterns in terms of miRNAs modulation in colorectal cancer, as well as bioactive dietary components able to modify gene expression through changes in miRNA expression. Furthermore, we emphasize on protective components such as resveratrol, curcumin, quercetin, α-mangostin, omega-3 fatty acids, vitamin D and dietary fiber, with a focus on the molecular mechanisms in the context of prevention and even treatment. In addition, several bioactive dietary components that have the ability to re-sensitize treatment resistant cells are described.

## 1. Introduction

World Health Organization estimates that about 30% of cancer deaths are attributed to modifiable risk factors like dietary habits and lifestyle factors such as physical inactivity, smoking and alcohol consumption [1]. Colorectal cancer (CRC) is the third most common type of cancer in the world, with a mortality rate exceeding 50% from that of incidence [2] and a higher incidence in developed countries [2,3]. Epidemiological studies have highlighted the link between colorectal cancer and its risk factors [4,5,6], including high consumption of red and processed meat [7], quality of dietary fatty acids [8], refined sugars, alcoholic beverages [9] and low consumption of foods containing dietary fiber [6,9,10,11]. Special focus has been given to red and processed meat (beef, pork, lamb, sausage, hamburger, etc.), especially in terms of culinary techniques [12]. Thus, cooking meat at high temperatures can cause the formation of harmful compounds such as polycyclic amines and polycyclic aromatic hydrocarbons [13] linked with colon carcinogenesis [14]. Moreover, other red meat-related mechanisms involved in CRC development are based on the formation of *N*-nitroso compounds from nitrite and ROS (Reactive Oxygen Species) from heme iron in the stomach. Both *N*-nitroso compounds and ROS can promote CRC development through increased cell proliferation and DNA damage [12,14].

Healthy dietary habits and particular natural components from food, along with an active lifestyle can prevent, slow and even reverse some disease processes particularly those associated with colorectal cancer. Several studies suggest that bioactive dietary components such as curcumin, resveratrol or quercetin act as chemopreventive agents in colorectal cancer [15,16,17], however the precise molecular mechanisms are still undeciphered and represent an effervescent research area. 

MicroRNAs (miRNAs or miRs) are a class of small, single-stranded, non-coding RNAs (~22 nucleotides long) that regulate gene expression at the post-transcriptional level [18,19]. These evolutionarily conserved molecules act by binding with perfect or imperfect complementarity to the 3′-untranslated region (3′-UTR) of a target messenger RNA (mRNA) resulting in degradation or inhibition of translation [19]. MiRNAs have been implicated in all biological processes including all stages of carcinogenesis, from initiation to tumor promotion and progression, influencing cell proliferation, differentiation, apoptosis, angiogenesis and metastasis [20,21]. It is thought that each miRNA can target several mRNAs and each mRNA can be targeted by more miRNAs, emphasizing the extremely important regulatory role of these molecules [22]. Furthermore, miRNA can act either as oncomirs (onco-miRNA) or tumor-suppressing miRNA. By their up-regulation, onco-miRNAs can reduce the expression of tumor suppression genes, while the down-regulation of tumor-suppressing miRNAs can increase the expression of oncogenes. 

A growing body of recent research has been focused on the importance of diet and biological dietary components for chemoprevention as well as treatment, based on miRNA modulation. Previous studies have demonstrated that bioactive food components can affect miRNA expression and thus their pathways, in many diseases, including cancer [23,24,25]. Colorectal cancer represents one of the most significant diet-related cancers. In this review, we describe how dietary patterns and bioactive dietary components can modify miRNA expression in colorectal cancer and therefore influence carcinogenesis, tumor progression and treatment. To our knowledge, this is the first review to emphasize the importance of miRNA regulation by dietary agents in colorectal cancer.

## 2. Biogenesis of miRNA

MiRNA synthesis takes place in the cell nucleus where miRNA are transcribed by RNA polymerase II into a hairpin-shaped primary transcript (pri-miRNA), which is hundreds to thousands of nucleotides in length [26,27]. The pri-miRNA will become a regulatory miRNA after several steps of processing stages. First, the pri-miRNA is processed into smaller transcripts (~70 nucleotides long) named pre-miRNA, by a microprocessor complex including DGCR8 (DiGeorge syndrome critical region 8) and RNA polymerase III Drosha [28,29,30]. The pre-miRNA is then carried out from the nucleus to the cytoplasm through nuclear pores by the nuclear export factor Exportin 5 [31,32].

**Figure 1:**
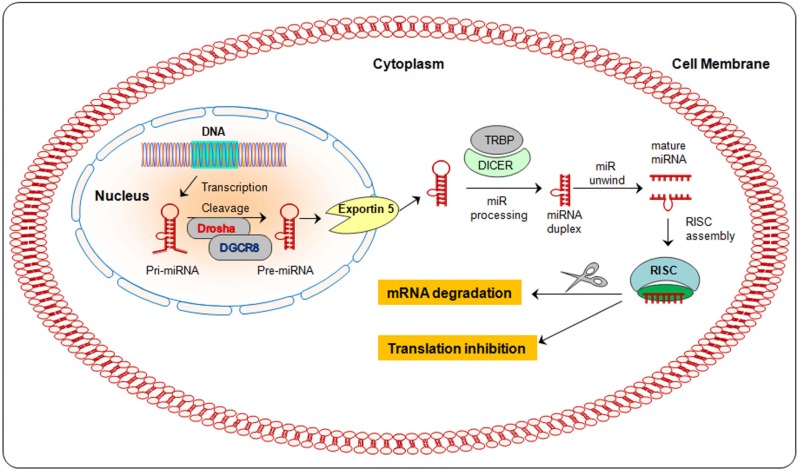
The biogenesis of miRNAs. MiRNAs are initially transcribed from introns or intergenic regions of DNA as long primary miRNA transcripts (pri-miRNA) and cleaved in pre-miRNAs smaller transcripts (~70 nucleotides long). After their export to the cytoplasm, pre-miRNAs are processed, unwind to mature miRNAs and loading into the RNA-induced silencing complex (RISC). This complex actively binds to mRNA targets and negatively regulates their gene expression and/or translational repression.

In the cytoplasm, the pre-miRNA is processed by Dicer (a helicase with RNase motif) to generate a mature miRNA of 21–23 nucleotides in length [27]. MicroRNAs negatively regulate the activity of mRNA of all protein-coding genes at post transcriptional level [33]. Generally, miRNAs bind through imperfect complementarity at the 3′UTR of specific mRNA target sequence [34,35], leading to the inhibition of translation and/or its degradation [36] (Figure 1). Recent evidence suggests that miRNAs can be packed into exosomes and transferred to other cells through the bloodstream, producing remote effects [36,37]. 

In normal cells, the expression of miRNAs is precisely controlled both for biogenesis and function, while, in tumor cells, miRNAs are abnormally expressed and processed. The genomic location of miRNAs is diverse, including introns (~40%), exons (~30%), uncertain transcriptional regions (~30%) and genomics repeats (~20%) [38]. In a previous study, Calin et al. [39] demonstrated that miRNA genes are frequently located at fragile sites that can be subjected to a variety of mechanisms including deletions, amplifications or mutations. These alterations modify the expression profile of miRNAs related to specific miRNA loci and therefore alter the expression of their mRNA target genes and also the mechanism they regulate. Moreover, even if the expression of miRNA is normal but mutations or a single-nucleotide polymorphism will occur in its sequence, the mRNA regulatory role can be altered [40,41]. A second mechanism responsible for aberrant expression of miRNAs is related to alterations in processing machinery, such as mutations in the Drosha and Dicer genes. Low expression of these genes was associated with advanced tumor stage and poor clinical outcome [42]. Epigenetic regulation represents the third mechanism responsible for aberrant regulation of miRNA expression. Hypermethylation represents one of the most important mechanisms that causes loss of tumor-suppressor miRNA expression. Thereby, by hypermethylation and/or deletion, the expression of miR-34b and miR-34C was downregulated in 90% of colorectal cancers, and was associated with oncogenesis [43]. Restoring the expression of tumor-suppressor miRNAs and/or blocking the expression of onco-miRNAs could be considered as alternative approaches for cancer prevention and treatment. 

## 3. Colorectal Cancer and miRNA

Discovery of miRNA and their utility as molecular tools in diagnosis, prognosis and therapy of CRC has opened lately countless research possibilities. MicroRNAs, through their action of oncomir and/or tumor-suppressing gene, are involved in all stages of CRC development and progress including carcinogenesis, progression, cell proliferation and angiogenesis [44,45,46]. Overproduction of miRNA arises as a response of amplification, translocation, pleomorphism or mutation in miRNA transcribing genes, whereas mutation, deletion, promoter methylation or any abnormalities in the miRNA biogenesis results in silencing of miRNAs in tumor cells [44,47]. MiRNAs that are up-regulated in tumor cells might act as oncomirs by down-regulating tumor suppressor genes. 

Extensive research effort has been conducted recently to investigate the potential of using miRNAs as biomarkers for diagnosis, tumor stage as well as for predicting patients’ outcome [48,49,50]. MiRNAs present unique features such as high tissue specificity, good sensitivity and stability. These molecules can also be identified in blood serum, as they are secreted in membrane vesicles as exosomes, in other body fluids, and in the stool sample [51,52]; therefore, they become promising candidates for ideal noninvasive biomarkers. Important miRNAs considered diagnostic biomarkers are: miR-29a, miR-221, miR-13-3p and miR-92a, found in plasma and miR-17-92 cluster; and miR-135, miR-92a, miR-21, miR-143, miR-154, miR-106a and miR-144 present in the stool sample [46,53].

The link between miRNAs and CRC progression has been described previously in mechanistic studies presenting that important proteins involved in key signaling pathways of CRC, such as Wnt/β-catenin, phosphatidylinositol 3-kinase (PI3K), KRAS, tumor protein 53 (p53) that are influenced by miRNA expression [47]. For example, EGFR/MAPK activation via KRAS down-regulation is strongly associated with let-7, miR-18a* and miR-143 [54,55,56]. In addition, PI3K pathway is activated by miR-126 [57], whereas miR-21 up-regulation leads to PI3K inactivation [58]. As a consequence, these pathways lead to increased cell survival, cell proliferation and initiation of angiogenesis. In addition, involved in the progression of adenoma to carcinoma is the CRC-specific miR-17-92 cluster by up-regulation of c-myc [59]. Chang et al. reported p53, a well-known apoptotic gene and of major importance in transformation of adenoma to carcinoma as a direct target of miR-34a [60]. Furthermore, modified miRNA expression can also be correlated to tumor stages and can regulate important factors associated with the metastatic cascade [53]. For example, the level of miR-31 is significantly higher in stage IV colorectal tumors as compared to stage II, while miR-21 expression is higher in metastatic than in non-metastatic CRC [61]. In addition, miRNAs are now investigated as therapeutic agents being excellent candidates due to their ability to target multiple genes and therefore restore important molecular pathways altered during cancer initiation and progression [47]. However in its early research stage, miRNA gene therapy presents great potential but there are still major challenges to unravel such as safe delivery to target tissue and possible side effects. The role of miRNA expression in colorectal cancer development and diagnostic is extensively described as well as manipulation strategies for miRNA gene therapy [37]. The present paper will further discuss the ability of diet and dietary components to modulate miRNA expression in colorectal cancer, offering possible explanations for the importance of dietary choices in order to prevent or even treat colorectal cancer.

## 4. Dietary Patterns and miRNA

The link between dietary patterns and colorectal cancer has been comprehensively described in literature [5,62,63], but the gene-regulator effect of energy balance and cancer pathways is yet an active field to be explored. As a consequence of an unbalanced diet and sedentary behavior, excess body mass represents a major risk factor for CRC. Obesity, along with abdominal fat distribution is a result of chronic positive energy balance and is strongly associated with colorectal cancer [63,64]. A first mechanism may be related to the role of the lipid storage, acting as a major endocrine organ secreting adipokine. Excess production of leptin and plasminogen activator inhibitor-1 (PAI-1) is associated with a decrease in adiponectin secretion that occurs in obese subjects. This condition contributes to abnormality cellular growth and stimulation of angiogenesis [65]. Caloric restriction, on the other hand, has been shown to reduce adiponectin [66] and has been inversely associated with colon cancer risk and progression [67]. In addition, a state of chronic over-nutrition may activate the oncogenes due to elevated levels of ROS [68]. Furthermore, obese subjects present a chronic condition with low-grade inflammation, caused by proteins and inflammatory cytokines, known as promoters of carcinogenesis [69]. These processes have been found to modify the expression of miRNAs such as let-7, miR-27 and miR-143 linked to obesity and cancer [70,71]. In addition, miR-143, which has been linked with adipogenesis [72], also has reduced expression in colorectal cancer [61]. Moreover, Olivo-Marston et al. [73] designed the first in vivo study in which they compared the effects of diet-induced obesity (DIO) vs. caloric restriction (CR) on colon carcinogenesis and explored energy balance interventions and microRNA expression in a murine colon cancer model. They successfully demonstrated that the DIO regimen resulted in an increase of colon tumor development, while the CR decreased it. In addition, DIO mice had significantly highest levels of insulin-like growth factor-1 (IGF1) along with elevated levels of cytokines including tumor necrosis factor-α (TNF-α) and interleukin-6 (IL-6) known to activate NF-κB (nuclear factor kappa-light-chain-enhancer of activated B cells). Furthermore, DIO down-regulated miR-150, known to promote apoptosis and decrease proliferation [74]. In addition, miR-155 and miR-196 known to be up-regulated in colorectal cancer [75] were up-regulated following DIO diet. Thereby, diet-induced obesity altered several biological pathways previously hypothesized to be involved in obesity and cancer.

Colorectal carcinogenesis has been closely linked with several features of the Western lifestyle. A typical Western diet is characterized by high intake of red and processed meats, refined starches, sugar and fat, along with poor intake of nutritious food groups such as fruits, vegetables, whole grains and healthy fatty acids [76]. Zhu et al. conducted an in vivo colon carcinogenesis experiment and were able to demonstrate that tumor promotion by a high-fat diet (20% fat vs. 5% fat for standard diet) needed active EGFR signals in the wild type EGFR mice. In addition, they showed that this dietary pattern can down-regulate tumor suppressor miRNA-143 and miRNA-145 through EGFR signaling, which further up-regulated their target oncogenes, MYC and KRAS, resulting in increased tumorigenesis [77].

Excessive intake of red and processed meat, alone or as part of a Western lifestyle has been positively associated with CRC development. One prospective study of sporadic CRCs with APC mutations showed that APC aberrations, especially GC to AT transitions were associated with increased intake of red processed meat due to hem and nitrites [78]. In addition, in a randomized cross-over trial, a high red meat (HRM) diet was shown to alter miRNA levels in rectal mucosa tissue. The miR-17-92 cluster and miR-21, both oncomirs known to be up-regulated in CRC, have been up-regulated following an HRM diet [79].

In contrast, a Mediterranean dietary pattern (MD) typically based on high intakes of anti-oxidant-rich foods like fresh fruits and vegetables, extra virgin olive oil, nuts, whole grains and fish lowered the risk of CRC [80]. Evidence confirmed the protective role of MD in the incidence and mortality of gastrointestinal cancers including CRC [81]. Furthermore, in vitro studies demonstrated that basic components of MD such as polyphenols, capsaicin, lycopene and resveratrol exert various anti-cancer properties on important pathways involved in colorectal carcinogenesis, thus promote apoptosis and cell growth inhibition [81,82]. In addition, DASH (Dietary Approaches to Stop Hypertension) dietary pattern was also inversely associated with risk of CRC in a large cohort of a postmenopausal woman [83] 

## 5. Bioactive Dietary Components and miRNA

**Figure 2:**
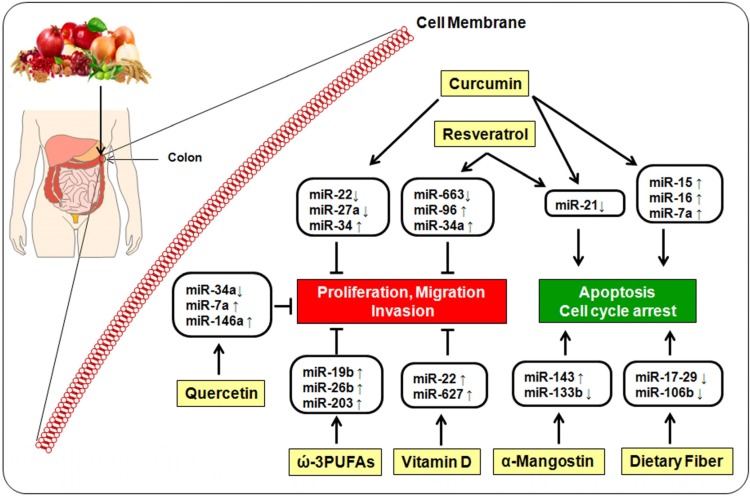
The protective role of bioactive dietary components in colorectal cells. Molecular mechanisms such as proliferation migration, invasion and apoptosis are tightly controlled by miRNAs-based epigenetic mechanisms.

Natural agents such as curcumin, resveratrol, quercetin, α-mangostin, ω-3-polyunsaturated fatty acids, vitamin D and dietary fiber have been reported to modulate the expression of miRNAs, affecting proliferation, migration, invasion and apoptosis (Figure 2).

### 5.1. Curcumin

Curcumin is a polyphenolic compound derived from turmeric (rhizomes of *Curcuma* *longa*), known as “Indian saffron”. Curcumin is the main ingredient in curry and is considered a bioactive dietary compound with anti-inflammatory, antioxidant, antimicrobial and anticarcinogenic properties [84]. Recent research highlighted the potential of curcumin in the prevention and therapy of cancer. Curcumin has the ability to inhibit cell proliferation and induce apoptosis by modulating the main proapoptotic pathways [17,85].

Anti-inflammatory properties of curcumin are due in part to the ability to inhibit COX-2, an enzyme involved in the inflammatory process and generation of inflammatory stimuli such as: nitric oxide synthase, NF-κB and prostaglandin E2 [86,87,88]. Kunnumakkara et al. [89] suggest that curcumin can enhance the effects of radiation therapy, inhibiting cell proliferation and angiogenesis, in colorectal cancer, by suppression of the NF-κB and their targeted genes. Curcumin can also inhibit cell growth by modulating Akt/mTOR pathway via EGFR downregulation [90,91].

The proapoptotic mechanism of curcumin, has been previously reported in colorectal cancer cells. Curcumin can up-regulate proapoptotic proteins of B-cell lymphoma-2 (Bcl-2) family such as Bax, Bak, Bim and Bid as well as the apoptotic protease activating factor-1 (Apaf-1) [92]. Likewise, it also sensitizes cells by inducing the oligomerization of Bax which favors the release of *cyt c* from mitochondria and activates caspase 3 and 9 [87,92]. By activating p53 gene and reducing TNF-α levels, curcumin counteracts survivin and IGF-1 antiapoptotic pathways resulting in apoptotic signal activation [93]. In other cell models curcumin can favor apoptosis by downregulating Bcl-2 and Bcl-XL antiapoptotic proteins by modifying miR-21, miR-15a and miR-16 expression [94,95,96]. In addition, curcumin treatment of esophageal cancer cells up-regulated tumor suppressor let-7a, which in turn affects Bax, Bcl-2 and caspase-3 [95].

An initial study by Sun et al. has shown for the first time that the biological effects of curcumin can be attributed in part to its potential to modulate miRNA [97]. Treatment of pancreatic cells with curcumin resulted in up-regulation of 11 miRNAs and downregulation of 18 miRNAs. In addition, up-regulation of miR-22 led to the specific suppression of Sp1 transcription factor (SP1) and estrogen receptor 1 (ESR1) suppression, respectively [97].

A well-known oncomir, miR-21, overexpressed in various cancers including colorectal cancer, modulates the expression of PTEN (phosphatase and tensin homolog) and PDCD4 (Programmed cell death-4) genes involved in cell proliferation and apoptosis [58]. In HCT116 cells, curcumin down-regulated miR-21 in a dose-dependent manner via transcription factor activator protein-1 (AP-1) transcription factor, inducing the expression of the tumor suppressor PDCD4. The same study showed in vivo effects of curcumin, suppressing cell proliferation, tumor growth, invasion and metastasis [98] (Table 1).

The miR-34 cluster (miR-34a, miR-34b, and miR-34c) which is down-regulated in colorectal cancer [99], may contribute to disease progression and drug resistance. Roy et al. studied difluorinated-curcumin, a synthetic analog of curcumin with improved bioavailability and effectiveness in re-expression of miR-34 suggesting its therapeutic potential in colorectal cancer (Table 1) [100]. In addition, another recent study presented curcumin’s ability to increase the therapeutic effect of 5-Fluorouracil in patients with treatment failure, confirming its ability to re-sensitize the resistance of CRC cells. In the same study, SP1, SP3, and SP4 were down-regulated by curcumin in SW-480 cells and this was accompanied by suppression of miR-27a and induction of zinc finger protein (ZBTB10) [101].

### 5.2. Resveratrol

Resveratrol is a potent polyphenol found in grapes skin, wine, berries and other plant sources. It is known for various health benefits such as antioxidant, anti-inflammatory, chemopreventive and antiviral properties [102]. One of the anti-inflammatory mechanisms for resveratrol is based on the inhibition of synthesis and releasing of pro-inflammatory factors as COX-2, counteracting NF-κB pro-inflammatory mechanisms [103]. Resveratrol can also regulate apoptosis and cell proliferation by increasing the expression of proapoptotic genes and down-regulating the expression of the antiapoptotic ones [104]. In colon cancer cells, resveratrol induces apoptosis by enhancing p53 levels and p21 in a p53 dependent and independent manner [104,105]. It also activates caspases 3 and 8 and increases Bax while decreasing Bcl-2 [16]. Some oncogenic mechanisms of CRC cells, including IGF-R1/PI3K/Akt and Wnt/B-catenin pathways, can also be suppressed by resveratrol [105,106]. Mir-21, a modulator of IGF-R1/PI3K/Akt pathway was previously demonstrated to be down-regulated by resveratrol treatment [107,108]. 

In a miRNA microarray study on SW480 human colon cancer cells, Tilli et al. identified a set of 46 miRNAs modulated by resveratrol [109]. The most important miRNAs down-regulated by resveratrol and known to behave as oncomiRs are listed in Table 1. Furthermore, In Silico analysis suggested that those miRNAs targeting PDCD4, PTEN and Dicer are important anti-proliferative factors. Moreover, resveratrol can down-regulate TGFβ1 in both miR-663-dependent and miR-663-independent manners. The expression of TGFβ1 in tumor and plasma was found to be significantly higher in patients with colorectal cancer and was correlated with tumor stage [110]. In a recent study on a genetically engineered mouse model for sporadic CRC it has been shown that resveratrol-treated mice presented increased levels of miR-96 compared with controls, associated with down-regulation of its KRAS target [111].

When combined with 5-Fluorouracil, resveratrol synergistically induce the growth inhibition and apoptosis, in colon cancer DLD-1 cells, by activating the MAPK/ERK1/2 signaling pathway. Moreover, resveratrol promotes growth inhibition by up-regulation of miR-34a, which down-regulate in cascade the E2F3 target gene and its downstream Sirt1 target [112].

### 5.3. Quercetin

Quercetin is a flavonoid found in many plants and foods such as onions, red wine, green tea and apples. It is a natural compound known to have a role in preventing carcinogenesis in colon cancer cells through anti-inflammatory as well as proapoptotic mechanisms. Quercetin inhibits COX-1 and COX-2 gene expression and down-regulates Bcl-2 through NF-κB inhibition [113,114]. In HT-29 colon cancer cells, quercetin reduces tumor volume and induces apoptosis both by AMP-activated protein kinase (AMPK) signaling via up-regulation of p53 [115] and by increasing the generation of intracellular ROS in a p53-independent manner [116]. Moreover, quercetin can decrease cell growth in SW480 cells by inhibiting cyclin D1 and survivin expression as well as regulating Wnt/β-catenin signaling pathway [117,118]. In addition, it can induce an antiproliferative effect in human CX-1 colon adenocarcinoma cells by suppressing hypoxia inducible factor-1α (HIF-1α) accumulation and a reduction of vascular endothelial growth factor (VEGF) secretion [119]. In HepG2 cancer cells, anti-tumor effects of quercetin were modulated by miR34a, mainly through the p53 related pathway [120] and let-7 can be a target of VEGF as a result from a computational model [121].

Noratto el al. reported that treatment of colon cancer cells with a flavonol-rich fraction of Yampon holly herb (Ilex vomitoria) containing quercetin, resulted in up-regulation of miRNA-146a [122]. This finding can explain in part the anti-inflammatory effect of quercetin by down-regulation of NF-κB via miR-146a. Moreover, in HT-29 colon cancer cells, treatment with quercetin in combination with resveratrol resulted in decreased SP1, SP3 and SP4 proteins, transcription factors known to be overexpressed in colon cancer. In addition, resveratrol and quercetin in combination induced ZBTB10 via miRNA-27a down regulation [123]. This study has opened a wide spectrum of research opportunities regarding the potential synergistic effects of bioactive dietary compounds to modulate microRNAs in colon cancer.

### 5.4. α-Mangostin

α-Mangostin is a polyphenolic xanthone derivative found in the pericarp of mangosteen fruit (*Garcinia mangostana*), a tropical fruit originated from Southeast Asia. Previous studies have shown that xanthones possess important biological properties such as antioxidant, anti-tumor, anti-inflammatory, chemopreventive and chemotherapeutic properties [124,125]. 

The anti-carcinogenic activity of α-mangostin may be due in part to its potential to inhibit oncogenic pathways such as Wnt/β-catenin [126], PI3K/Akt and MAPK/ERK1/2 [112]. In human colon cancer colo 205 cells, α-mangostin induced cytotoxic effects in a dose and time-dependent manner and induced apoptosis through extrinsic and intrinsic pathways including expression of caspase-3,-8,-9, release of cytocrome c from mitochondria and up-regulation of Bax, Bmf, Bak, Bid and p53 [127]. Another study, on HT-29 colon cell lines and its xenograft, demonstrated anti-tumorigenic activity of α-mangostin via down-regulation of antiapoptotic protein Bcl-2 and β-catenin. Moreover, lower levels of transcription factors such as c-myc, c-jun and cyclin D1, were observed in reduced tumor mass, of mice fed with a diet containing α-mangostin [128]. 

In an attempt to identify new molecular targets modulated by α-mangostin, Nakagawa et al. [129] evaluated the cytotoxic effect of α-mangostin on DLD-1 cells, and found, along with the cytotoxic effect mainly due to apoptosis, elevated levels of miRNA-143, which negatively regulates Erk5 translation. Furthermore, Kumazaki’s group observed that intracellular level of miR-133b was down-regulated after treatment of DLD-1 cells with α-mangostin, whereas its target gene death receptor5 (DR5) was up-regulated. DR5 represents a target for tumor necrosis factor related apoptosis-inducing ligand resistant (TRAIL), and was transferred from the cytoplasm to the tumor cell surface membrane. This evidence strongly indicated that α-mangostin can function as a sensitizer of TRAIL-induced apoptosis [130]. Additionally, the combination treatment of DLD-1 cells with α-mangostin and 5-Fluorouracil both at 2.5 μM resulted in a greater growth inhibition compared with the treatment of alone 5 μM of α-mangostin or 5-Fluorouracil respectively [129].

### 5.5. ω-3-Polyunsaturated Fatty Acids

ω-3-Polyunsaturated fatty acids (ω-3PUFAs) are protective agents in various human cancers including colorectal cancer. The protective role of diets rich in ω-3PUFAs, containing walnuts, fish oil, soybeans and seed oil, against colon carcinogenesis has been demonstrated in clinical, epidemiological and laboratory animal-based studies [131,132], whereas, ω-6PUFA rich diets based on sunflower oil, corn oil or soybean oil, have been proved to enhance the development of colon tumors [133].

Previous studies reported that ω-3PUFAs induce apoptosis through mitochondrial-mediated pathways, including loss of mitochondrial membrane potential, generation of ROS, accumulation of intracellular Calcium(2+), activation of caspase 3 and caspase 9 and increase in the Bax/Bcl-2 expression ratio [134]. In addition, it was shown that docosahexaenoic acid (DHA), a member of the omega-3 fatty acid family, modulates apoptotic pathways such as PI3K and p38/MAPK [135,136]. Let-7, the well-known highly conserved miRNA family can regulate important proteins such as previously described Bcl-2, MAPK and caspase as a response to diets high in fatty acids [137]. In human colon adenoma LT97 cell line, the treatment with eicosapentaenoic acid (EPA) and DHA lowered the levels of Bcl-2 a stronger effect being observed for EPA compared with DHA. Neither EPA nor DHA had any effect on HT29 cells [138].

Recent research suggested that the chemopreventive activity of ω-3PUFAs originated from fish oil may be in part due to modulation of intestinal miRNA. In this regard, Davidson et al. [132] evaluated the effects of ω-3PUFAs on miRNA expression in the colon of rats injected with azoxymethane (a colon-specific carcinogen). They reported that ω-3PUFA enriched diet modulates miRNA expression in the colon. Specifically, expression of five miRNAs (let-7d, miR-15b, miR-107, miR-191, miR-324-5p) was not affected by azoxymethane treatment in the fish oil fed group. In addition, the chemopreventive effect of ω-3PUFAs is greater when fermentable fiber (pectin) rather than poorly fermentable (cellulose) is added to the diet [132,139]. Their further research showed that the fish oil + pectin diet leads to up-regulation of miR-19b, miR-26b and miR-203 following down-regulation of their oncogenic targets including IGF1 receptor, IGF2 receptor and transcription factor 4 [140]. These important findings underline the significant role of diet as a key factor for prevention of carcinogenesis.

### 5.6. Vitamin D

Vitamin D exerts a protective role in colorectal cancer as shown by epidemiological studies, which link low vitamin D diet or circulating level of calcidiol (25-hydroxyvitamin D_3_) with increased risk of colorectal cancer [141,142]. Mechanistic studies have shown that vitamin D (1,25(OH)_2_D_3_) can antagonize Wnt signaling in human colon cancer cells in multiple ways [143] and influences inflammatory pathways involved in cancer progression such as COX-2 and NF-κB [144]. 

It has been reported that vitamin D regulates miRNA expression in colon cancer cells. Dias et al. identified miR-22 as a target of 1,25(OH)_2_D_3_ in human colon cancer cells and showed that can mediate in part its inhibitory effect on cell proliferation and migration [145]. Another recent study has demonstrated that 1,25(OH)_2_D_3_ up-regulates miR-627 and contributes to the anticancer properties of vitamin D in colon cancer by targeting histone demethylase JMJD1A [146], thereby inhibiting proliferation of colon cancer cells in vitro and in vivo through epigenetic regulation. This mechanism may explain at least partially the protective action of vitamin D on colorectal cancer but this still remains an active area of research.

### 5.7. Dietary Fiber

A growing body of evidence suggests that high consumption of food containing dietary fiber has a protective effect against colorectal cancer, confirmed by systematic review evidence which identified that 10 g fiber intake per day decreases the risk of colorectal cancer by 10% [147]. One mechanism through which high fiber diets decrease the risk of colon cancer is most likely due to increased production of butyrate by fermentation of dietary fiber in the intestine. Butyrate which is a short-chain fatty acid produced by dietary fiber fermentation in the colon is known as a chemoprotective agent [148]. 

In HT29 and HCT116 CRC cells butyrate was shown to significantly decrease the expression of the miR-17-92 oncogenic cluster which led to upregulation of its target genes including PTEN, Bcl-2L11 and CDKN1A [149]. In addition, the same group of researchers demonstrated that in vivo supplementation of a high red meat diet (300 g/day lean red meat) with butylated resistant starch (40 g/day butylated high amylose maize starch), restored miR-17-92 levels to baseline, thus miR-21 remained unchanged [79]. Recently, a novel mechanism of butyrate able to induce apoptosis and decrease proliferation was described [150]. Along with miR-17-29 down-regulation previously described, Hu et al. demonstrated that butyrate decreases c-myc and increases p57 expression in colon cancer cells. In addition, butyrate induces expression of p21, a key regulatory molecule of cell cycle arrest by miR-106b downregulation [151]. Collectively, this data provide mechanistic insights proving that antiproliferative and proapoptotic activity of butyrate may be in part explained by changes in miRNA activity.

A summary of bioactive dietary components involved in miRNA modulation, along with molecular targets and biological effects are presented in Table 1.

### 5.8. Other Dietary Factors

Recent studies have revealed that along with the bioactive dietary components described in this paper, there are other foods rich in bioactive agents able to change miRNA expression in CRC. For example, miRNA profiles of azoxymethane-injected rats are modified by diet. Intake of polyphenol-rich pomegranate juice resulted in elevated levels of miR-126 [152], whereas grape seed extract rich in flavonoids modulate several miRNAs among which miR-135b, miR-196a and miR21 are known to be up-regulated in CRC [153]. Another recent research found that miRNA expression profiles were changed after walnut intake [154], mostly due to its content in protective fatty acids. Thereby, miR-1903, miR-467c, miR-3068 and miR-297a modulated by walnuts affect both target genes that are involved in blocking of inflammation, angiogenesis and proliferation, and genes that are involved in promoting of apoptosis. Furthermore, dysregulation of let-7/c-Myc/Lin27 axis in heterocyclic amine-induced colon carcinogenesis might be partially reversed by dietary spinach [25]. 

As presented earlier in this review, miRNAs are likely great candidates as diagnostic biomarkers as well as therapeutic agents. Bioactive dietary agents have been demonstrated to exert a protective effect against colon carcinogenesis as confirmed by their activity on important miRNAs know as CRC biomarkers (Table 1). 

## 6. Conclusions

There is increasing interest in using dietary interventions to prevent, slow and even reverse chronic disease, especially cancer. Decades of observations and epidemiological studies suggested that our everyday food choices influence the risk of cancer. Epigenetic changes and molecular alterations which link nutrition to carcinogenesis is an active area of research, including emerging data indicating that diet and bioactive dietary components can modify gene expression through miRNA modulation.

Nutrition factors and dietary patterns influence colorectal cancer risk. The majority of colorectal cancer cases is sporadic and thought to be preventable through diet, which remains an equally important link during and after treatment. We highlighted in this review the newest data on dietary modulation of miRNA in colorectal cancer. Western dietary patterns can modify miRNA expression linking obesity with cancer. However, there are healthy dietary patterns such as MD and DASH inversely associated with CRC risk. The majority of bioactive dietary components presented in this paper (curcumin, resveratrol, quercetin, ω-3 PUFA, α-mangostin, and vitamin D) are thought to be powerful tools in colorectal cancer prevention and treatment due to their ability to change miRNA expression, thereby modulating important pathways involved in cell proliferation, tumor growth, apoptosis, invasion and metastasis. Considering the extensive benefit in terms of miRNA regulation by bioactive dietary agents and their potential to down-regulate oncomirs (miR-21, miR-17-92 cluster, miR-92, etc.), activate tumor suppressor genes (p53, PDCD4, and PTEN) and re-sensitize treatment-resistant cells, we strongly believe that in the near future, dietary based miRNA manipulation can be used along with other forms of anticancer therapy. Further knowledge from mechanistic studies able to identify molecular targets and signaling pathways modulated by miRNA–diet interaction are still challenging. Although extensive research of both, nutrition and cancer, is still needed until results from in vitro or animal studies can be translated in everyday diet, the potential benefit in terms of cancer prevention and even treatment will be worth our time and patience.

## Figures and Tables

**Table 1:** Examples of bioactive dietary components that modulate miRNA expression: molecular targets and biological effects.

Dietary Component	miRNA Modulated by Dietary Compounds	Molecular Target	Biological Effect	References
Curcumin Curcumin (synthetic analog (CDF))	↓miR-21	PDCD4	Cell cycle arrest, invasion, metastasis	[72]
	↑miR-34a, ↑miR-34c	Notch-1	Apoptosis, cell proliferation	[74]
	↓miR-27a	Sp1, Sp3, Sp4, ZBTB10	Cell growth, angiogenesis, inflammation	[75]
Resveratrol	↑miR-663, ↓miR-17, ↓miR-21, ↓miR-25, ↓miR-92a-2, ↓miR-103-1, ↓miR-103-2	TGF-β1, PDCD4, PTEN, Dicer	Cell proliferation	[83]
	↑miR-34a	E2F3	Growth inhibition	[86]
	↑miR-96	KRAS	Chemoprevention, tumor growth	[85]
Quercetin Quercetin + Resveratrol	↑miR-146a	NF-kβ	Inflammation	[96]
	↓miR-27a	Sp1, Sp3, Sp4, ZBTB10	Cell growth, angiogenesis, inflammation	[97]
α-mangostin	↑miR-143	ERK-5	Apoptosis	[102]
	↓miR-133b	DR5	Apoptosis	[103]
ω-3 PUFA	* miR-15b	Bacel, Serbp1	Plasminogen Activation	[105]
	* miR-107	Bcl-2, CCNE1	Apoptosis, Cell cycle	
	* miR-191, * miR324-5p, * let-7d	-	-	
ω-3 PUFA + soluble fiber (pectin)	↑miR-19b, ↑miR-26b, ↑miR-203	IGF1R, IGF2R, TCF4	Cell proliferation, migration	[113]
Vitamin D	↑miR-627	JMJD1A	Cell proliferation	[119]
	↑miR-22	NELL2, OGN, HNRPH1, RERE, NFAT5	Cell proliferation, migration	[118]
Fiber (butyrate)	↓miR-17-92	PTEN, Bcl-2L11, CDKN11A	Cell proliferation, apoptosis	[122,123]
	↓miR-106b	p21	Cell cycle arrest	[125]

* Not affected in the presence of natural agent when exposed to a colon-specific carcinogen; Abbreviations: Bcl-2, B-cell lymphoma-2; EGFR, epidermal growth factor receptor; DR5, death receptor 5; ERK, Extracellular signal-regulated protein kinases; IGF1/2R, Insulin-like growth factor-1/2 receptor; JMJD1A, Jumanji domain containing 1A; KRAS, V-Ki-ras2 Kirsten rat sarcoma viral oncogene homolog; miR, microRNA; NF-κB, nuclear factor κB; p21, protein 21; PDCD4, Programmed cell death4; PTEN, phosphatase and tensin homolog; Sp, specificity protein; TCF4, transcription factor 4; TGFβ, Transforming growth factor beta; TNF-α, Tumor necrosis factor-α; ZBTB10, zinc finger protein.
